# All-Trans Retinoic Acid Enhances Bacterial Flagellin-Stimulated Proinflammatory Responses in Human Monocyte THP-1 Cells by Upregulating CD14

**DOI:** 10.1155/2019/8059312

**Published:** 2019-12-26

**Authors:** Thi Xoan Hoang, Jong Hyeok Jung, Jae Young Kim

**Affiliations:** Department of Life Science, Gachon University, Seongnam, Gyeonggi-Do 461-701, Republic of Korea

## Abstract

All-trans retinoic acid (ATRA), an active form of vitamin A, exerts immunomodulatory functions. In this study, we examined the immune potentiating effect of ATRA on bacterial flagellin-induced NF-*κ*B activation and proinflammatory cytokine production in human monocytic cell line THP-1. ATRA treatment significantly enhanced the flagellin-induced NF-*κ*B/AP-1 activity in THP-1 via the RAR/RXR pathway. Similarly, ATRA enhanced the expression and production of TNF-*α* and IL-1*β* in THP-1 cells upon flagellin challenge. The cell surface expression of toll-like receptor 5 (TLR5), which is the receptor for bacterial flagellin, was significantly reduced by ATRA in a concentration- and time-dependent manner. To determine the mechanisms underlying the ATRA-enhanced immune response against bacterial flagellin despite the reduced cell surface expression of TLR5 in ATRA-treated THP-1, we examined the cell surface expression of CD14, which has been proposed to be a TLR co-receptor that enhances the response to microbial components. The cell surface expression of CD14 was significantly enhanced by ATRA treatment, especially in the presence of flagellin. Anti-CD14 antibody treatment prior to ATRA and flagellin treatments completely abolished ATRA-enhanced TNF-*α* and IL-1*β* production. Our results suggest that ATRA enhances flagellin-stimulated proinflammatory responses in human monocyte THP-1 cells by upregulating CD14 in a RAR/RXR-dependent manner.

## 1. Introduction

It has long been known that vitamin A is essential for resistance to infection and that vitamin A deficiency can lead death due to infection [1]. All-trans retinoic acid (ATRA), an active form of vitamin A, plays an important regulatory role in cell growth and differentiation [2] by regulating target gene expression through the action of two transcription factors, retinoid acid receptor (RAR) and retinoid X receptor (RXR) [3]. ATRA also plays critical roles in the immune system such as induction of T cell migration into mucosal sites [4], generation and homing of IgA-secreting B cells [5], induction of regulatory T cells [6], and regulation of effector CD4^+^ T cell differentiation and function [7, 8]. Because of its important role in the immune system, it is hypothesized that ATRA may enhance the immune responses of monocytes/macrophages against bacterial components. However, several previous in vitro studies have reported that ATRA treatment suppresses the immune responses of monocytes/macrophages against bacterial products. ATRA inhibited proinflammatory cytokine production in mouse macrophages stimulated with bacterial lipopolysaccharide (LPS), phorbol 12-myristate 13-acetate (PMA), or IFN-*γ* [9, 10]. Similarly, ATRA suppressed the synthesis of IL-12 and TNF-*α*, but enhanced the production of IL-10 in LPS-stimulated human monocytic cell line THP-1 and human cord blood mononuclear cells [11]. In another study, ATRA decreased the proinflammatory cytokine production of primary human monocytes stimulated with bacterial lipopeptide (TLR2/1 ligand) by reducing TLR2 expression [12]. In addition, ATRA significantly inhibited LPS-induced prostaglandin E2 production and cyclooxygenase-2 expression in mouse peritoneal macrophages, as well as TNF-*α* release in rat peripheral blood mononuclear cells [13]. To the best of our knowledge, only one study has reported on the immune potentiating effects of ATRA, which increases the flagellin-mediated immune response of human monocytes by inducing TLR5 expression [14].

In this study, we aimed to confirm the possible immune potentiating effect of ATRA on human monocytes/macrophages by investigating the bacterial flagellin-induced NF-*κ*B activation and proinflammatory cytokine production in THP-1 cells.

## 2. Materials and Methods

### 2.1. Cell Culture

The human monocytic THP-1 cells (ATCC, Manassas, VA, USA) were grown in RPMI-1640 (Welgene Inc., Gyongsan, Korea) supplemented with 10% heat-inactivated fetal bovine serum (FBS, Invitrogen, Gibco BRL, MD, USA), 1% antibiotic-antimycotic (Invitrogen), 10 mM HEPES buffer (Invitrogen), and *β*-mercaptoethanol (Sigma-Aldrich, St. Louis, MO, USA) at 37°C in a 5% CO_2_ humidified incubator. To cultivate THP1-XBlue cells (InvivoGen, San Diego, CA, USA), RPMI-1640 medium containing 10% heat-inactivated FBS, 1% antibiotic-antimycotic, and 200 *μ*g/ml Zeocin (InvivoGen) was used.

### 2.2. NF-*κ*B/AP-1 Activation Reporter Assay

To measure NF-*κ*B activation, THP1-XBlue reporting cells, which express the embryonic alkaline phosphatase gene under control of NF-*κ*B and AP-1 ([Supplementary-material supplementary-material-1]), were cultured in 24-well plates at 0.8 × 10^6^ cells/well or 96-well plates at 2 × 10^5^ cells/well. To determine the effect of ATRA (Sigma-Aldrich) on the NF-*κ*B activation of THP-1 cells against bacterial flagellin, we used two different types of flagellins from *S. typhimurium* and *B. subtilis*, which were purchased from InvivoGen. ATRA was prepared as a 20 mM stock in dimethyl sulfoxide (DMSO) and stored at −20°C before being diluted to the desired concentration in medium in each experiment. To determine agonist effects, RAR*α* agonist BMS753 and RXR*α* agonist LG100268, which were purchased from Sigma-Aldrich, were used. Cells were treated with agonist for 24 h and then stimulated with 10 or 100 ng/ml of flagellin from *S. typhimurium* for 24 h. We chose concentrations of ATRA and flagellin according to established protocols [14, 15]. To determine antagonist effects, RAR*α* antagonist BMS195614 (Sigma-Aldrich) and RXR*α* antagonist UVI 3003 (Santa Cruz Biotechnology, Dallas, Texas, USA) were used. Cells were preincubated with 1 *μ*M antagonists for 2 h, followed by treatment with 1 *μ*M ATRA for 24 h and then stimulation with 10 and 100 ng/ml of flagellin from *S. typhimurium* for 24 h. An aliquot of the flagellin-stimulated culture supernatant was added to QUANTI-Blue alkaline phosphatase detection medium (InvivoGen) for 2 h color development at 37°C. Absorbance was measured at 630 nm using an ELISA microplate reader (*μ*-Quant; Bio-Tek Instruments, Winooski, USA).

### 2.3. Quantitative Real-Time Polymerase Chain Reaction (PCR)

Cells were treated with 1 *μ*M ATRA or DMSO for 24 h and then stimulated with flagellin from *S. typhimurium* for 4 h. Total RNA extraction was performed using a Qiagen RNAeasy mini kit (Qiagen, Hilden, Germany) according to the manufacturer's instructions. RNA concentrations were determined with a MaestroNano Microvolume spectrophotometer (Maestrogen, Las Vegas, NV, USA). cDNA synthesis was performed by reverse transcription using Hyperscript RT master mix (GeneAll, Seoul, Korea) with an Oligo (dT) primer (Invitrogen) and 2 *μ*g of total RNA at 42°C for 1 h. Quantitative real-time PCR was performed on the Rotor-gene system (Qiagen) using the Platinum SYBR Green qPCR SuperMix-UDG (Invitrogen). PCR amplification was performed using following primer sets: TNF-*α* 5′-tgagcactgaaagcatgatcc-3′, 5′-ggagaagaggctgag gaaca-3′; IL-1*β* 5′-gggataacgaggcttatgtgc- 3′, 5′-aggtggagagctttcagttca-3′; and *β*-actin 5′-caccattggcaatga gcggttc-3′, 5′-aggtctttgcggatgtccacgt-3′. Normalization of target gene expression levels was performed using the human *β*-actin gene as an endogenous control. For each sample, the relative abundance of target mRNAs was calculated from the C_Δ*t*_ values of the target and *β*-actin genes based on the 2^−ΔΔ^cycle threshold (Ct) method.

### 2.4. Flow Cytometry

To investigate the surface expression of TLR5 and CD14, cells were seeded onto a 25T tissue culture flask at 3 × 10^6^ cells and treated with ATRA and flagellin from *S. typhimurium*. Treated cells were collected and divided into 1 × 10^6^ cells per tube and then washed twice with phosphate-buffered saline (PBS), followed by incubation with a phycoerythrin-conjugated anti-TLR5 (85B152.5, Imgenex, Sorrento Valley, CA, USA) or anti-CD14 (My4, BD Biosciences Pharmingen, San Diego, CA. USA) antibody at 4°C in the dark for 30 min. After washing with PBS, cells were resuspended in PBS before analysis on a Cytomics FC500 MLP (Beckman Coulter, Fullerton, CA, USA). Forward and side-scatter plots were used to exclude dead cells and debris from the plots of histogram analysis and proportion of dead cells was less than 5%.

### 2.5. Enzyme-Linked Immunosorbent Assay (ELISA)

THP-1 cells were seeded into 25T cell culture flasks at 3 × 10^6^ cells and preincubated with or without ATRA for 24 h. Then, cells were stimulated with 10 or 100 ng/ml of flagellin for 24 h. The supernatant was collected from the flagellin-stimulated cultures. Quantification of secreted IL-1*β* and TNF-*α* was performed using IL-1*β* (Biolegend, San Diego, CA, USA) and TNF-*α* platinum ELISA kits (eBioscience, San Diego, CA, USA) according to the manufacturer's instruction. Absorbance measurements were obtained using an ELISA microplate reader at 450 nm.

### 2.6. Statistical Analysis

Statistical significance was assessed by Student's *t* test or one-way analysis of variance (ANOVA) using SPSS 12.0 for Windows. Tukey HSD (honestly significant difference test) was used for groups of data with equal variances or alternatively the Games–Howell test for groups with unequal variances in the post hoc test for ANOVA. Data are shown as mean ± standard deviation (SD). Differences were considered significant at *p* < 0.05.

## 3. Results

### 3.1. ATRA Enhances NF-*κ*B/AP-1 Activity of THP-1 Cells Exposed to Flagellins Obtained from *S. typhimurium* or *B. subtilis*

**Figure 1:**
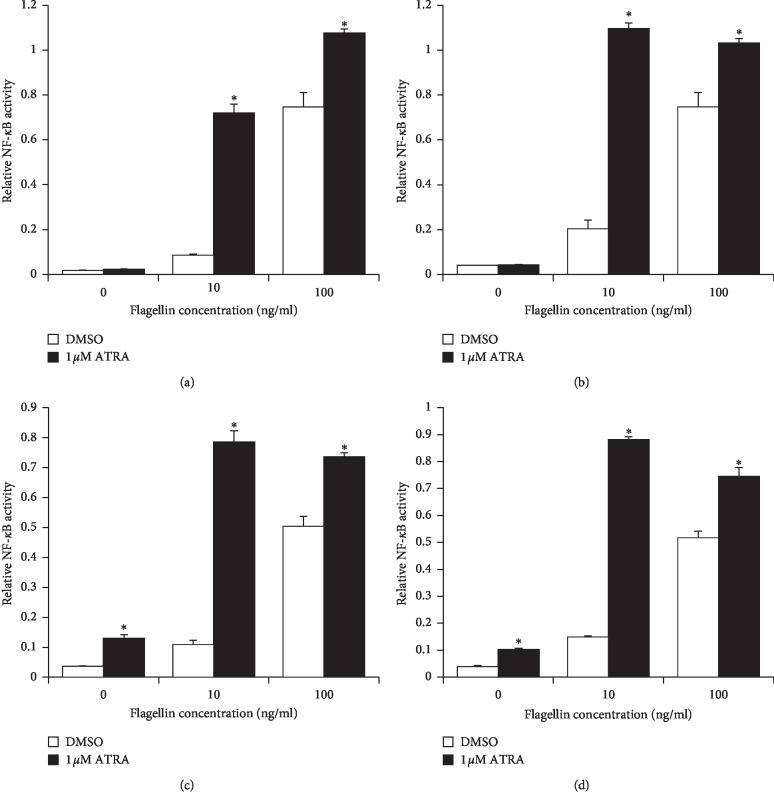
ATRA enhances NF-*κ*B/AP-1 activity in THP-1 cells upon flagellin challenge. THP1-XBlue cells were treated with 1 *μ*M ATRA or DMSO for 24 h (a), 48 h (b), and 72 h (c) and then stimulated with flagellin from *S. typhimurium* (a–c) or *Bacillus subtilis* (d) at various concentrations as indicated above for 24 h. Secreted alkaline phosphatase in cell culture supernatants was measured by ELISA to determine NF-*κ*B/AP-1 activity. Bar graphs indicate relative NF-*κ*B/AP-1 activity ± SD. Statistical significance was assessed by Student's *t* test using SPSS. ^*∗*^*p* < 0.05 vs. DMSO-treated groups.

To determine the effect of ATRA on the immune response of human monocytes against bacterial flagellin, we used two different types of flagellins from *S. typhimurium* and *B. subtilis* and examined NF-*κ*B/AP-1 activity using THP1-XBlue cells. Cells were treated with 1 *μ*M ATRA for 24–72 h and then stimulated with 10 or 100 ng/ml flagellin from *S. typhimurium* or *B. subtilis* for 24 h. ATRA treatment significantly enhanced the flagellin-induced NF-*κ*B/AP-1 activity of THP-1 cells, compared to that of the DMSO-treated control group. The maximum NF-*κ*B activity was observed at 24 h and 48 h after ATRA treatment in the 100 ng/ml and 10 ng/ml flagellin-treated groups, respectively (Figures 1(a)–1(c)). Because the molecular size and structure of flagellins from *B. subtilis* are known to be different from those from *S. typhimurium* [16], we examined the NF-*κ*B/AP-1 activity of THP-1 cells upon *B. subtilis*-derived flagellin challenge. A similar increase in NF-*κ*B/AP-1 activity was observed in ATRA-treated THP-1 cells upon *Bacillus* flagellin challenge (Figure 1(d)). The immune potentiating effects of ATRA on NF-*κ*B/AP-1 activity were found be much more profound in the cells treated with lower concentrations of 10 ng/ml flagellin than 100 ng/ml (Figures 1(a)–1(d)).

### 3.2. ATRA-Enhanced NF-*κ*B/AP-1 Activity of THP-1 Cells upon Flagellin Challenge Depends on RAR-RXR

**Figure 2:**
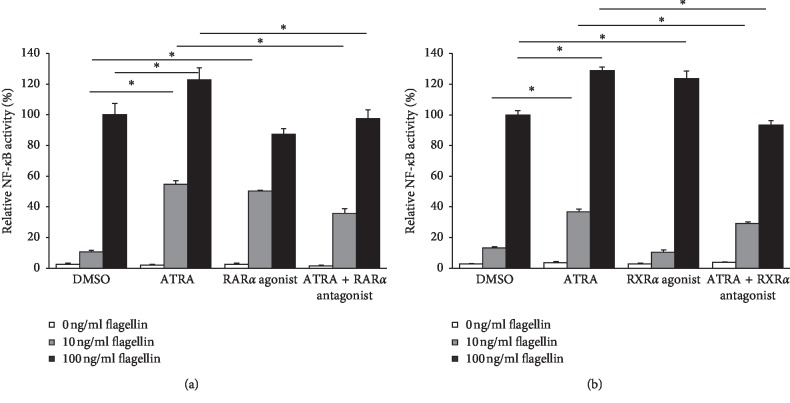
Effect of RAR*α*, RXR*α* agonist, or antagonist on NF-*κ*B/AP-1 activity of THP-1 cells upon flagellin challenge. To determine RAR/RXR agonist effects, THP1-XBlue cells were treated with 1 *μ*M RAR*α* agonist BMS753 (a) or 1 *μ*M RXR*α* agonist LG100268 (b) for 24 h and then treated with different concentrations of flagellin as indicated above for 24 h to determine RAR/RXR antagonist effects; cells were preincubated with 1 *μ*M RAR*α* antagonist BMS195614 (a) or RXR*α* antagonist UVI3003 (b) for 2 h and then treated with 1 *μ*M ATRA or DMSO for 24 h followed by stimulation with different concentrations of flagellin from *S. typhimurium* as indicated above for 24 h. Secreted alkaline phosphatase in cell culture supernatants was measured by ELISA to determine NF-*κ*B/AP-1 activity. Bar graphs indicate relative NF-*κ*B/AP-1 activity ± SD. Statistical significance was assessed by one-way ANOVA using SPSS. ^*∗*^*p* < 0.01.

To determine whether the retinoic acid receptor pathway mediates the ATRA-induced enhancement of NF-*κ*B/AP-1 activity in THP-1 cells upon flagellin challenge, we performed an experiment using agonists and antagonists of RAR*α* and RXR*α*, which are representative nuclear receptors of the RAR-RXR heterodimer signaling pathway [17]. Similar to treatment with 1 *μ*M ATRA, 1 *μ*M RAR*α* agonist alone significantly enhanced the NF-*κ*B/AP-1 activity of THP-1 cells upon 10 ng/ml *S. typhimurium* flagellin challenge but not upon 100 ng/ml flagellin challenge (Figure 2(a)). The treatment with 1 *μ*M RAR*α* antagonist partially reversed the effect of ATRA on the cells exposed to 10 ng/ml flagellin, while it completely reversed that on cells exposed to 100 ng/ml flagellin (Figure 2(a)). However, treatment with 1 *μ*M RXR*α* agonist alone only enhanced the NF-*κ*B/AP-1 activity of THP-1 cells exposed to 100 ng/ml flagellin but not to 10 ng/ml flagellin (Figure 2(b)). The treatment with 1 *μ*M RXR*α* antagonist partially reversed the effect of ATRA on the cells exposed to 10 ng/ml flagellin but completely reversed that on cells exposed to 100 ng/ml flagellin (Figure 2(b)). Results suggest that the effect of ATRA on flagellin-induced NF-*κ*B activity is mediated by the canonical RAR-RXR pathway.

### 3.3. ATRA Enhances Expression and Production of TNF-*α* and IL-1*β* in THP-1 Cells upon Flagellin Challenge

**Figure 3:**
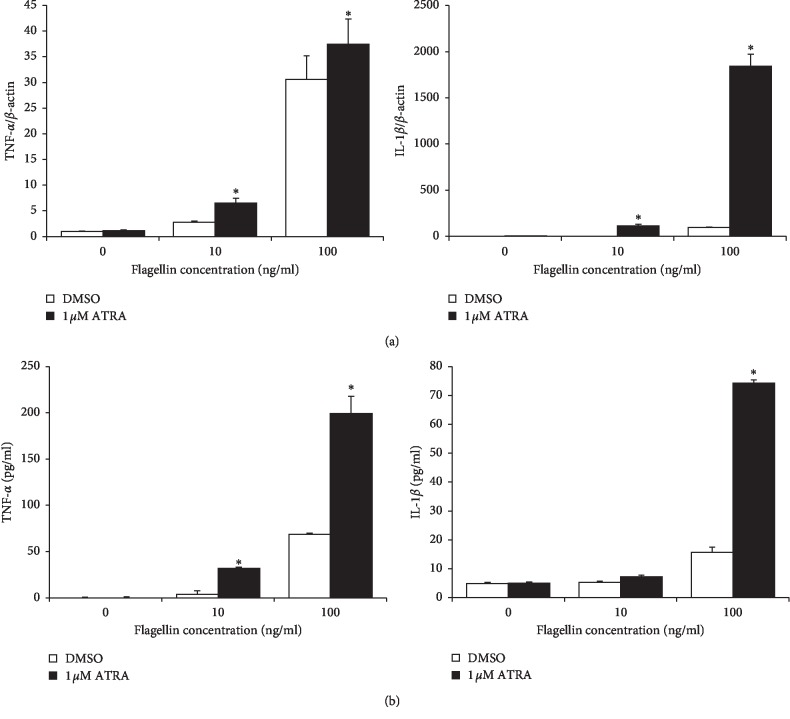
ATRA enhances the expression and secretion of proinflammatory cytokines in THP-1 cells upon flagellin challenge. (a) THP-1 cells were treated with 1 *μ*M ATRA or DMSO for 24 h and then stimulated with flagellin from *S. typhimurium* for 4 h mRNA expression levels of IL-1*β* and TNF-*α* were measured by RT-qPCR. Bar graphs indicate relative mRNA expression ± SD. (b) THP-1 cells were treated with 1 *μ*M ATRA or DMSO for 24 h and then stimulated with flagellin from *S. typhimurium* for 24 h; the cell culture supernatant was collected and subjected to ELISA to determine TNF-*α* and IL-1*β* production. Bar graphs indicate secreted cytokine amount ± SD. Statistical significance was assessed by Student's *t* test using SPSS. ^*∗*^*p* < 0.01 vs. DMSO-treated groups.

**Figure 4:**
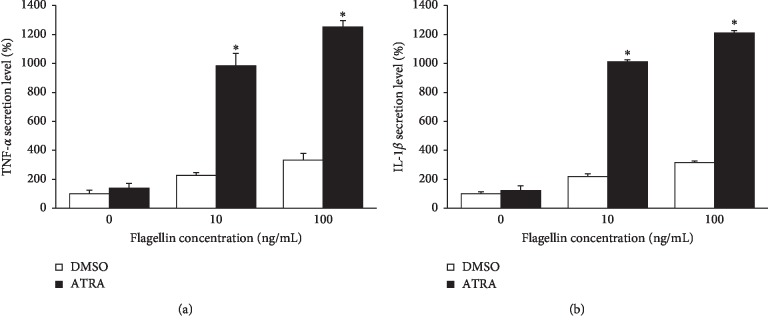
ATRA enhances the production of proinflammatory cytokines in THP-1 cells upon *Bacillus subtilis* flagellin challenge. THP-1 cells were treated with 1 *μ*M ATRA or DMSO for 24 h and then stimulated with flagellin from *B. subtilis* for 24 h; the cell culture supernatant was collected and subjected to ELISA to determine the production of TNF-*α* (a) and IL-1*β* (b). Bar graphs indicate secreted cytokine amount ± SD. Statistical significance was assessed by one-way ANOVA using SPSS. ^*∗*^*p* < 0.05 vs. DMSO-treated group.

To determine the possible immune potentiating effects of ATRA, we treated THP-1 cells with 1 *μ*M ATRA for 24 h, followed by stimulation with flagellin for 24 h. Then, the cellular expression and production of TNF-*α* and IL-1*β* were examined. Consistent with the increase in NF-*κ*B/AP-1 activity, the mRNA expression of TNF-*α* and IL-1*β* was enhanced by ATRA (Figure 3(a)). ATRA increased the mRNA expression of IL-1*β* approximately 1,800-fold compared to the DMSO control (Figure 3(a)). Moreover, ATRA also significantly enhanced the protein production of TNF-*α* and IL-1*β* compared with the DMSO control group (Figure 3(b)). To prove that purchased flagellin is not contaminated with LPS, we performed an experiment with polymyxin B, which is a potent inhibitor of LPS contamination. Our data demonstrated that the phenomena seen in our study is not due to LPS contamination since the presence of polymyxin B did not affect the level of TNF-*α* and IL-1*β* secretion from flagellin-stimulated THP-1 cells ([Supplementary-material supplementary-material-1]). We also examined the effect of ATRA on TNF-*α* and IL-1*β* production in *B. subtilis* flagellin-stimulated THP-1 cells. ATRA enhanced TNF-*α* and IL-1*β* production in *B. subtilis* flagellin-stimulated THP-1 cells, similar to that in *S. typhimurium* flagellin-treated cells (Figure 4). Taken together with the results of NF-*κ*B activation, these results indicate that ATRA enhances inflammatory responses against bacterial flagellin.

### 3.4. ATRA Reduces Cell Surface TLR5 Expression of THP-1 in Concentration- and Time-Dependent Manners

**Figure 5:**
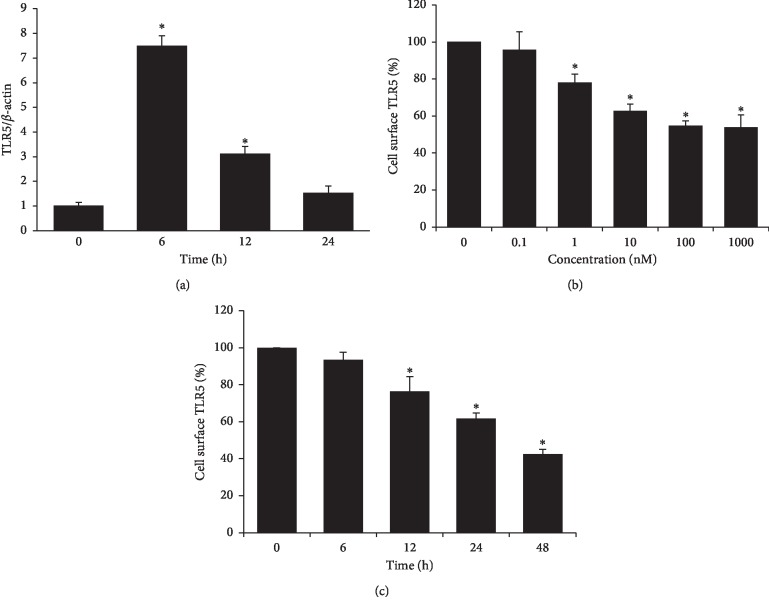
ATRA upregulates TLR5 mRNA but decreases cell surface TLR5 expression of THP-1 cells in a time- and concentration-dependent manner. THP-1 cells were treated with 1 *μ*M ATRA for different time periods as indicated above (a, c) or with various concentrations of ATRA as indicated above for 24 h (b). The mRNA level was assessed by qRT-PCR (a) and cell surface expression of TLR5 was measured by flow cytometry (b, c). Bar graphs indicate relative TLR5 expression ± SD. ^*∗*^*p* < 0.05 vs. DMSO control.

To determine the mechanism for the ATRA-induced enhancement of NF-*κ*B/AP-1 activity and proinflammatory cytokine production in THP-1 cells upon flagellin challenge, we examined the effects of ATRA on the mRNA or cell surface expression of TLR5, which is the corresponding bacterial flagellin receptor. As shown in Figure 5(a), TLR5 mRNA expression increased sharply after 6 h of treatment with 1 *μ*M ATRA and then gradually decreased. In contrast to the mRNA expression, the cell surface expression of TLR5 in THP-1 cells started to decrease upon 24 h-treatment with as low as 1 nM ATRA and it gradually decreased with ATRA concentration (Figure 5(b)). The cell surface expression of TLR5 was gradually reduced in a time-dependent manner upon treatment with 1 *μ*M ATRA, and the expression level at 48 h after ATRA treatment was approximately 45% of that of the control (Figure 5(c)). The present results suggest that the ATRA-enhanced inflammatory responses against flagellin in THP-1 cells may not be due to the increased cell surface levels of TLR5, opposing the findings of the previous report which indicated that ATRA enhances cell surface expression of TLR5 in THP-1 cells [14].

### 3.5. ATRA Enhances Cell Surface CD14 Expression of THP-1 and Anti-CD14 Ab Treatment Reverses Flagellin-Induced TNF-*α* and IL-1*β* Production of ATRA-Stimulated THP-1

**Figure 6:**
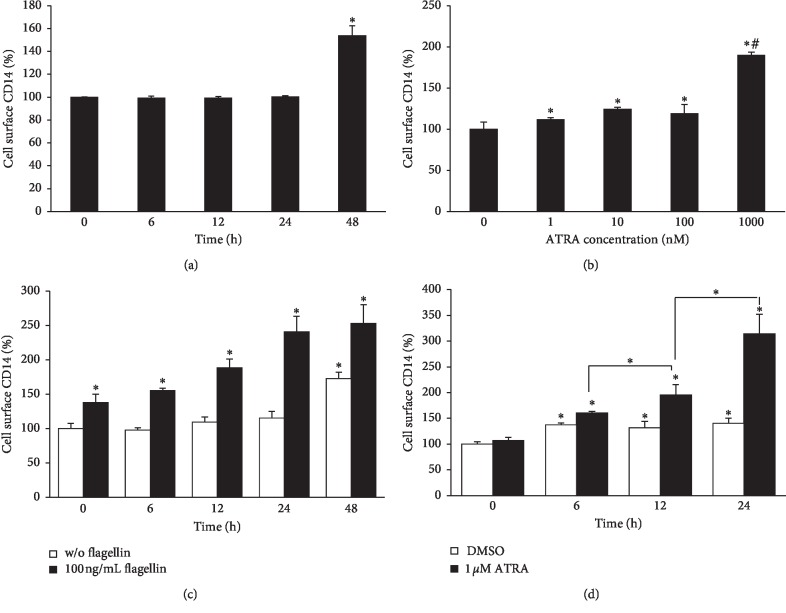
ATRA enhances the cell surface CD14 expression in THP-1 cells. Cells were treated with 1 *μ*M ATRA for different time points (a) or with different concentrations of ATRA for 48 h (b). Cells were treated with 1 *μ*M ATRA for different time periods as indicated and followed by challenge with 100 ng/ml flagellin from *S. typhimurium* for 24 h (c). Cells were treated with 1 *μ*M ATRA for 24 h and then subjected to 100 ng/ml flagellin from *S. typhimurium* for different time periods as indicated (d). Cell surface expression of CD14 was measured by flow cytometry. Bar graphs indicate relative fluorescence intensity ± SD. Statistical significance was assessed by one-way ANOVA using SPSS. ^*∗*^*p* < 0.05 vs. DMSO control; ^#^*p* < 0.05 vs. 1–100 nM ATRA-treated groups (b).

**Figure 7:**
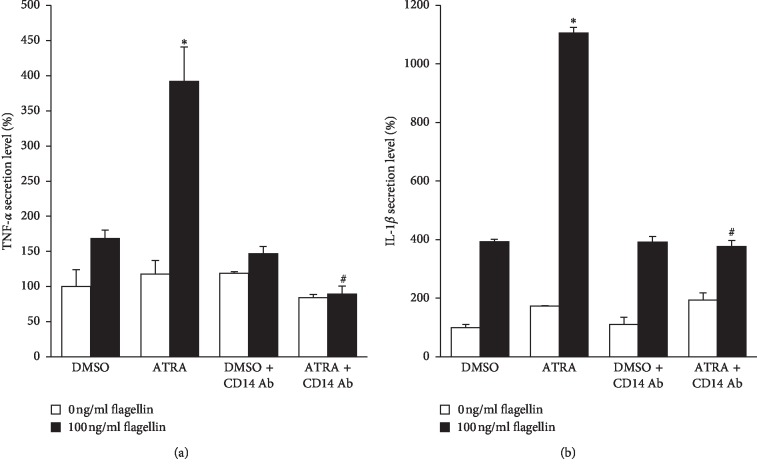
Anti-CD14 antibody treatment suppresses flagellin-induced TNF-*α* and IL-1*β* secretion of ATRA-treated THP-1 cells. (a) THP-1 cells were treated with 1 *μ*g/ml anti-CD14 antibody (My4) for 30 min prior to treatment with 1 *μ*M ATRA or DMSO for 24 h and then stimulated with 100 ng/ml of flagellin from *S. typhimurium* for 24 h; the cell culture supernatant was collected and subjected to ELISA for detecting TNF-*α* production. (b) THP-1 cells were treated with 1 *μ*g/ml anti-CD14 antibody (My4) for 30 min prior to treatment with 1 *μ*M ATRA or DMSO for 24 h and then stimulated with 100 ng/ml flagellin for 24 h; the cell culture supernatant was collected and subjected to ELISA detecting for IL-1*β* production. Bar graphs indicate secreted cytokine amount ± SD. Statistical significance was assessed by one-way ANOVA using SPSS. ^*∗*^*p* < 0.01 vs. DMSO control; ^#^*p* < 0.01 vs. ATRA group.

To determine the mechanism underlying the ATRA-induced enhancement of inflammatory responses upon flagellin challenge in THP-1 cells despite reduced cell surface TLR5 levels, we examined the cell surface expression of CD14, which is a co-receptor that enhances the response to microbial components induced by most TLRs [18]. As shown in Figure 6(a), the cell surface expression of CD14 was not changed in THP-1 cells until 24 h after treatment with 1 *μ*M ATRA. However, it was significantly enhanced at 48 h after ATRA treatment. CD14 expression levels of THP-1 cells treated with ATRA at concentrations ranging from 1 to 1000 nM for 48 h were higher than that of DMSO-treated cells (Figure 6(b)). ATRA alone in the absence of flagellin could induce cell surface CD14 expression only at 48 h after the treatment (Figure 6(c)). However, the addition of flagellin for 24 h after treatment with 1 *μ*M ATRA for different time periods significantly enhanced cell surface CD14 expression, and this enhancement was dependent on ATRA treatment time (Figure 6(c)). Flagellin alone in the absence of ATRA could enhance cell surface CD14 expression as early as 6 h following treatment, but this enhanced expression was not further increased over time (Figure 6(d)). Interestingly, pretreatment with ATRA for 24 h synergistically enhanced cell surface CD14 expression of flagellin-stimulated THP-1 cells over time (Figure 6(d)). These results clearly indicate that ATRA and flagellin synergistically enhance cell surface CD14 expression of THP-1 cells. To confirm that ATRA-enhanced surface CD14 expression is responsible for the ATRA-induced enhancement of proinflammatory cytokine production, we treated THP-1 cells with anti-CD14 antibody prior to ATRA and flagellin treatments. Results showed that anti-CD14 antibody treatment completely abolished ATRA-enhanced TNF-*α* and IL-1*β* production (Figure 7).

## 4. Discussion

In the present study, we demonstrated that ATRA treatment enhances NF-*κ*B activation and proinflammatory cytokine production in human monocytic cells upon bacterial flagellin challenge. These results may reflect how vitamin A contributes to the immune response of monocytes/macrophages against bacterial infection in vivo. ATRA can increase the cellular expression of NF-*κ*B subunits, p65 and p50 [19–22], and induce downstream genes by activating the NF-*κ*B transcription factor [23], which is one of the most important regulators of gene expression of proinflammatory cytokines such as TNF-*α* and IL-1*β* [24]. Thus, the ATRA-induced enhancement of the expression and production of proinflammatory cytokines in THP-1 cells, which was observed in our study, might be mainly mediated through the NF-*κ*B pathway. In addition, ATRA is known to bind RAR to form a heterodimer with RXR. Our results show that the RAR agonist alone could enhance NF-*κ*B activation, while the RAR antagonist alone could suppress ATRA-enhanced NF-*κ*B activation, suggesting that the ATRA-induced enhancement of NF-*κ*B activation depends on the classical RAR/RXR signaling pathway. However, because ATRA does not bind RXR, our observation that the RXR agonist alone is able to induce NF-*κ*B activation, which can be reversed by the RXR antagonist alone, also indicates a RAR-independent mechanism of NF-*κ*B activation. Because the RXR agonist, LG100268, could activate the RXR-PPAR*α*, RXR-PPAR*γ* heterodimer [25], and RXR homodimer [26], the activation of NF-*κ*B could have been mediated by the nonclassical signaling pathway.

Similar to our observations, Cho et al. reported that ATRA induces the expression of TNF-*α*, IL-1*β*, IL-12, several costimulatory molecules, and MHC molecules in flagellin-stimulated THP-1 cells [14]. They also showed that ATRA induces cell surface TLR5 expression in THP-1 cells, which is in contrast to our observation that ATRA suppressed cell surface TLR5 expression in THP-1 cells in a concentration- and time-dependent manner. It is uncertain why the cell surface TLR5 expression in THP-1 cells was decreased by ATRA treatment in the present study. However, other studies also reported ATRA-induced downregulation of TLR2 and TLR4 expressions in human monocytes [12] and primary rat epithelial cells [27], respectively; however, they did not elucidate the mechanisms underlying the effect.

In the present study, we investigated the cell surface expression of CD14, which is a co-receptor for several TLRs, to determine how the inflammatory response of THP-1 cells upon flagellin challenge was increased even though the cell surface expression of its corresponding receptor, TLR5, was significantly inhibited by ATRA treatment. Our results clearly demonstrated that ATRA and flagellin synergistically enhance cell surface CD14 expression of THP-1 cells. Similar to our results, several studies showed that ATRA increases CD14 expression in human monocyte-like U937 cells [28] and promyelocytic leukemia cell line NB4 [29, 30]. The CD14 molecule has been proposed to act as a co-receptor for TLR2 [31], TLR3 [32], TLR7, and TLR9 [33], in addition to its classical partner, TLR4 [34]. An efficient response to microbial components by most TLRs requires the activity of the co-receptor CD14, which efficiently delivers the microbial component to the TLR [35, 36] and amplifies TLR-mediated proinflammatory responses [33, 37–39]. Based on the results showing that the neutralization of CD14 molecules with monoclonal anti-CD14 antibody My4 completely reversed ATRA-enhanced pro-inflammatory cytokine production, we suggest that ATRA-enhanced CD14 expression is responsible for the ATRA-induced enhancement of proinflammatory cytokine production upon flagellin challenge and might compensate for the reduction of cell surface TLR5 expression in THP-1 cells. Indeed, a recent study has demonstrated that enhancing CD14 surface expression of dendritic cells can compensate the defect in endosomal TLR4 signaling [40].

In this study, the stimulatory effects of ATRA on NF-*κ*B activation and TNF-*α* expression and secretion were more pronounced at lower concentrations (10 ng/ml) of flagellin than at higher concentrations (100 ng/ml) (Figures 1 and 3). These phenomena may be explained by the fact that one of the critical functions of CD14 as a co-receptor for TLR4 is the reduction of LPS concentrations required to be detected by macrophages. Even at very low LPS concentrations, CD14 is able to bind to LPS and transfer it to the TLR4/MD2 complex and thus can enhance the sensitivity of macrophages in detecting infections by Gram-negative bacteria [41]. Based on this, we speculate that CD14 may reduce the concentrations of flagellin required to be detected by THP-1 cells via a similar mechanism of CD14 in LPS sensing, although there is no report on the interactions between CD14 and flagellin so far. In contrast, the stimulatory effect of ATRA on IL-1*β* expression and secretion was more pronounced in the presence of higher concentrations of flagellin (Figure 3). Unlike TNF-*α*, it is speculated that such compensating effects of CD14 are not exerted in IL-1*β* secretion. This is because IL-1*β* production and secretion require both transcriptional induction of pro-IL-1*β* by TLR5 signaling and cleavage of caspase-1 by several inflammasomes including NLRC4, which is a cytoplasmic receptor of flagellin [42], making unlikely to interact with cell surface CD14 molecules.

In conclusion, our study suggests that ATRA enhances bacterial flagellin-stimulated proinflammatory responses in human monocyte THP-1 cells by upregulating cell surface CD14 expression in a RAR/RXR-dependent manner.

## Data Availability

The data used to support the findings of this study are available from the corresponding author upon request.

## Abbreviations

ATRA: All-trans retinoic acid
RAR: Retinoic acid receptor
RXR: Retinoid X receptor
TLRs: Toll-like receptors
PMA: Phorbol 12-myristate 13-acetate
LPS: Lipopolysaccharide.

## Supplementary Materials

Supplementary Materials Figure S1: schematic diagram for mechanism of NF-*κ*B activation reporting assay using THP1-XBlue cells. Figure S2: ATRA-enhanced proinflammatory cytokine secretion upon flagellin challenge is not due to LPS contamination.
