# Supplementary material for: All-Trans Retinoic Acid Enhances Bacterial Flagellin-Stimulated Proinflammatory Responses in Human Monocyte THP-1 Cells by Upregulating CD14
Source: Biomed Res Int. 2019 Dec 26;2019:8059312. doi: 10.1155/2019/8059312 (PMC6948301; doi:10.1155/2019/8059312)
Supplement: Supplementary Materials — Figure S1: schematic diagram for mechanism of NF-κB activation reporting assay using THP1-XBlue cells. Figure S2: ATRA-enhanced proinflammatory cytokine secretion upon flagellin challenge is not due to LPS contamination. [file 8059312.f1.pdf]

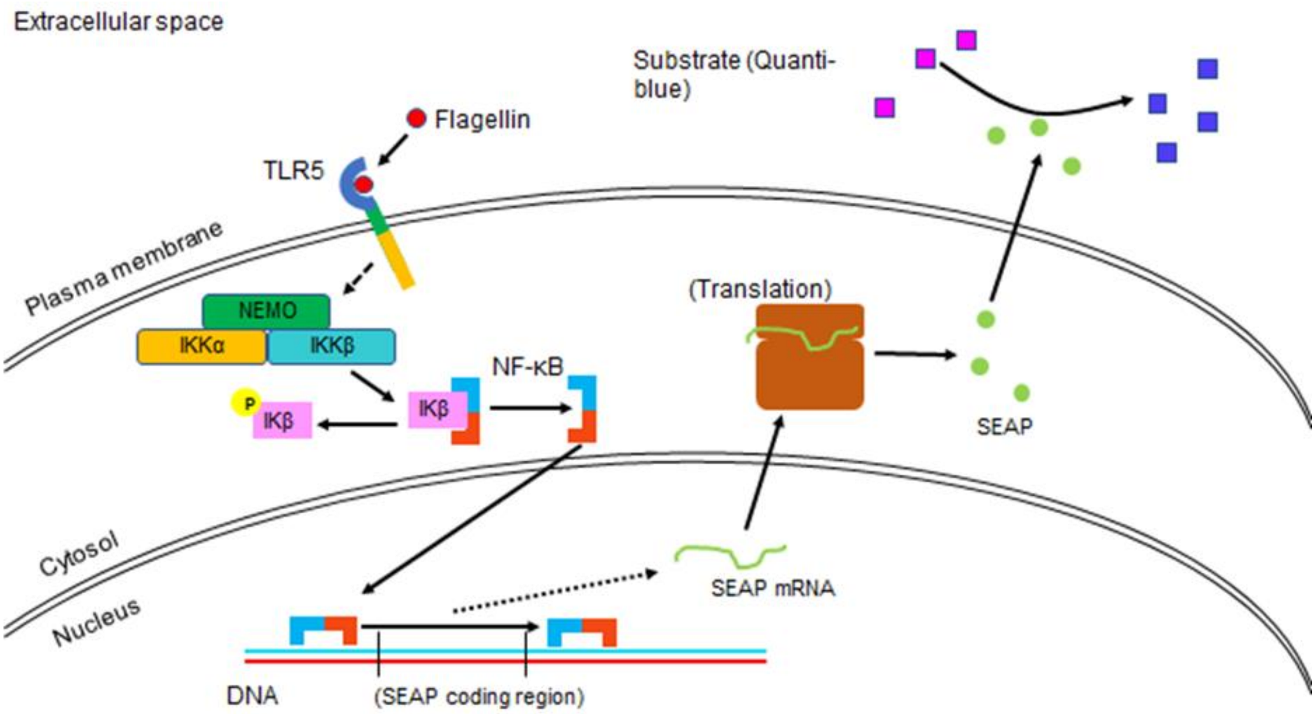

Figure S1. Schematic diagram for mechanism of NF-κB activation reporting assay using THP1-XBlue cells. THP-1 Xblue cells stably express NF-κB/AP-1 inducible secreted alkaline phosphate (SEAP) gene. Upon activation of Toll-like receptor pathway by specific ligands, such as bacterial flagellin, these cells are activated to make an intracellular signaling, which eventually activates transcription factor NF-κB, then SEAP is subsequently expressed. Secretion of SEAP is easliy detectable by specific substrate Quanti-blue, which turns blue in the presence of SEAP. The blue color development can be detected by ELISA reader. SEAP, secretion of embryonic alkaline phosphatase; NEMO, NF-κB essential modulator; IKK, IκB kinase.

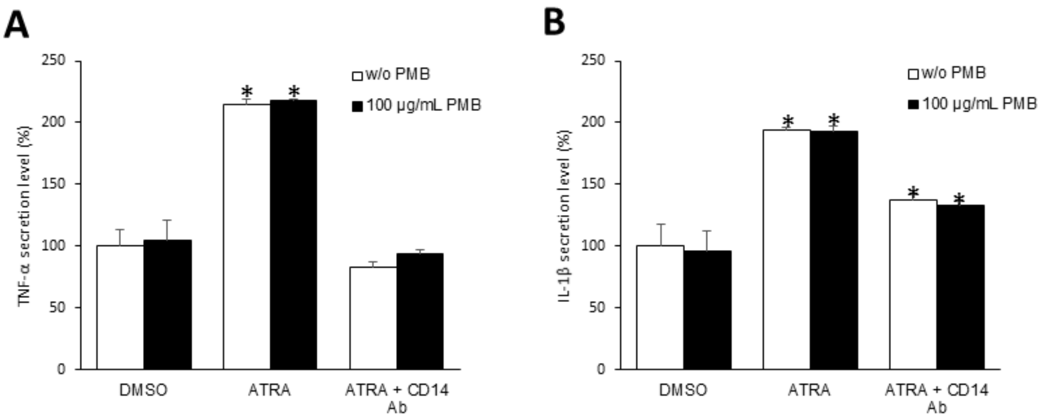

Figure S2. ATRA-enhanced proinflammatory cytokine secretion upon flagellin challenge is not due to LPS contamination. THP-1 cells were pretreated with 1 µg/mL CD14 antibody (My4) for 30 min prior to treatment with or without 1 µM ATRA for 24 h and then stimulated with flagellin from *S. typhimurium* in the presence of 100 µg/mL polymyxin B (PMB) for 24 h. Cell culture supernatant was collected and subjected to the ELISA assay for TNF-α (A) or IL-1β (B) to quantify amount of secreted cytokine. Bar graphs indicate cytokine secretion level ± SD. Statistical significance was assessed by one-way ANOVA using SPSS. * $p < 0.05$ vs DMSO group.
